# Etiology of respiratory tract infections in the community and clinic in Ilorin, Nigeria

**DOI:** 10.1186/s13104-017-3063-1

**Published:** 2017-12-07

**Authors:** Olatunji Kolawole, Michael Oguntoye, Tina Dam, Rumi Chunara

**Affiliations:** 1 UNILORIN Institute of Molecular Science and Biotechnology, Infectious Diseases and Environmental Health Research Group, University of Ilorin, Ilorin, Nigeria; 2 Kwara State Primary Health Care Development Agency, Ilorin, Nigeria; 3 Mailman School of Public Health, Columbia University, New York, USA; 4 Computer Science & Engineering and College of Global Public Health, New York University, New York, NY USA

**Keywords:** Nigeria, Respiratory infection, Community

## Abstract

**Objective:**

Recognizing increasing interest in community disease surveillance globally, the goal of this study was to investigate whether respiratory viruses circulating in the community may be represented through clinical (hospital) surveillance in Nigeria.

**Results:**

Children were selected via convenience sampling from communities and a tertiary care center (n = 91) during spring 2017 in Ilorin, Nigeria. Nasal swabs were collected and tested using polymerase chain reaction. The majority (79.1%) of subjects were under 6 years old, of whom 46 were infected (63.9%). A total of 33 of the 91 subjects had one or more respiratory tract virus; there were 10 cases of triple infection and 5 of quadruple. Parainfluenza virus 4, respiratory syncytial virus B and enterovirus were the most common viruses in the clinical sample; present in 93.8% (15/16) of clinical subjects, and 6.7% (5/75) of community subjects (significant difference, p < 0.001). Coronavirus OC43 was the most common virus detected in community members (13.3%, 10/75). A different strain, Coronavirus OC 229 E/NL63 was detected among subjects from the clinic (2/16) and not detected in the community. This pilot study provides evidence that data from the community can potentially represent different information than that sourced clinically, suggesting the need for community surveillance to enhance public health efforts and scientific understanding of respiratory infections.

## Introduction

Acute Respiratory Infections (ARIs) (the cause of both upper respiratory tract infections (URIs) and lower respiratory tract infections (LRIs)) are a major cause of death among children under 5 years old particularly in developing countries where the burden of disease is 2–5 times higher than in developed countries [1]. While these viruses usually cause mild cold-like symptoms and can be self-limiting, in recent years novel coronaviruses such as severe acute respiratory syndrome (SARS) and Middle East respiratory syndrome (MERS) have evolved and infected humans, causing severe illness, epidemics and pandemics [2]. Currently, the majority of all infectious disease outbreaks as recorded by the World Health Organization (WHO) occur in the continent of Africa where there is high transmission risk [3, 4]. Further, in developing areas (both rural and urban), there are increasing risk factors such as human-animal interfaces (due to residential-proximity to livestock). These changing epidemiological patterns have resulted in calls for improved ARI surveillance, especially in places of high transmission risk [5].

Nigeria is one such place with high prevalence of many of the risk factors implicated in ARI among children including; age, sex, overcrowding, nutritional status, socio-economic status, and where study of ARIs is currently limited [6]. These broad risk factors alongside limited resources have indicated the need for community-based initiatives for surveillance and interventions [6, 7]. For ARI surveillance in particular, infections in the community are those that do not get reported clinically. Clinical data generally represents the most severe cases, and those from locations with access to healthcare institutions. In Nigeria, hospitals are visited only when symptoms are very severe. Thus, it is hypothesized that viral information from clinical sampling is insufficient to either capture disease incidence in general populations or its predictability from symptoms [8]. Efforts worldwide including in East and Southern Africa have been focused on developing community-based participatory disease surveillance methods [9–13]. Community-based approaches have been shown useful for learning more about emerging respiratory infections such as assessing under-reporting [14], types of viruses prevalent in communities [10], and prediction of epidemics [15].

Concurrently, advancements in molecular identification methods have enabled studies regarding the emergence and epidemiology of ARI viruses in many locations (e.g. novel polyomaviruses in Australia [16, 17], human coronavirus Erasmus Medical Center (HCoV-EMC) in the Middle East and United Kingdom [18, 19], SARS in Canada and China [20–22]), yet research regarding the molecular epidemiology of ARI viruses in Nigeria is limited. Diagnostic methods available and other constraints have limited studies there to serological surveys of only a few of these viruses and only in clinical populations [23, 24]. Thus, the utility of community-based surveillance may be appropriate in contexts such as in Nigeria, and the purpose of this pilot study was to investigate if clinical cases may describe the entire picture of ARI among children in Nigeria.

## Main text

### Materials and methods

We performed a cross-sectional study in three community centers and one clinical in Ilorin, Nigeria. Ilorin is in Kwara state and is the 6th largest city in Nigeria by population [25]. Three Local Government Areas (Ilorin East, Ilorin South and Ilorin West LGAs) were the community sites and Children’s Specialist Hospital, Ilorin the clinical site. Convenience sampling was used for the purposes of this pilot study, and samples were obtained from March 28 to April 5 2017. Inclusion criteria were: children less than 14 years old who had visible symptoms of ARI within the communities or those confirmed at the hospital with ARI. Exclusion criteria were: children who were 14 and above, not showing signs of ARI and subjects whose parents did not give consent. Twenty-five children with symptoms were selected each from the three community locations while 16 symptomatic children were sampled from the hospital. The total sample size (n = 91) was arrived at based on materials and processing cost constraints, as well as to provide enough samples to enable descriptive understanding of viral circulation patterns estimated from other community-based studies [10].

### Measurements/data collection

Disease Surveillance and Notification Officers, who are employed by the State Ministry of Health and familiar with the communities in this study, performed specimen and data collection. Symptoms considered were derived in accordance with other ARI surveillance efforts: sore throat, fever, couch, running nose, vomiting, body ache, leg pain, nausea, chills, shortness of breath [10, 26]. Gender and age, type of residential area (rural/urban), education level, proximity of residence to livestock, proximity to an untarred road and number of people who sleep in same room, were all recorded. The general difference between the two settings was that those from the hospital had severe illnesses, while those from the community were generally “healthy” but exhibiting ARI symptoms (i.e. mild illness).

Nasal swabs were collected from the subjects and stored in DNA/RNA shield (Zymo Research, Irvine, California). Collected samples were spinned and the swab removed. Residues containing the nasal samples were stored at – 20 °C prior to molecular analysis.

### Specimen processing protocol

Viral RNA was isolated using ZR Viral RNA™ Kit (Zymo Research, Irvine, California) per manufacturer instructions (http://www.zymoresearch.com/downloads/dl/file/id/147/r1034i.pdf). Real-time PCR (polymerase chain reaction), commonly used in ARI studies [10, 19, 27], was then carried out using RV15 One Step ACE Detection Kit, catalogue numbers RV0716K01008007 and RV0717B01008001 (Seegene, Seoul, South Korea) for detection of 15 human viruses: parainfluenza virus 1, 2, 3 and 4 (PIV1–4), respiratory syncytial virus (RSV) A and B, influenza A and B (FLUA, FLUB), rhinovirus type A-C, adenovirus (ADV), coronavirus (OC 229 E/NL63, OC43), enterovirus (HEV), metapneumovirus (hMPV) and bocavirus (BoV).

Reagents were validated in the experimental location using an inbuilt validation protocol to confirm issues of false negative and false positive results were not of concern. Amplification reaction was carried out as described by the manufacturer: reverse transcription 50 °C-30′, initial activation 94°-15′, 45 cycles: denaturation 94°-30″, annealing 60°-1′ 30″, extension 72°-1, final extension 72°-10′, hold 4°. Visualization was performed using electrophoresis on a 2% agarose gel in TBE 1X with EtBr, in presence of RV15 OneStep A/B/C Markers; molecular weight marker. Specimen processing was not blinded as there was no risk of experimental bias. Standardized procedures were used for community and clinic sampling.

### Statistical analysis

All statistical analyses were performed using R version 3.2.4. Univariate statistics [mean and 95% confidence interval (CI)] are described. Bivariate statistics (difference in proportions) were assessed using a two-proportion z-test. A p value < 0.001 was considered significant. No observations used in this study had any missing data for analyses in this study.

### Results

Basic participant demographics are summarized in Table 1. In terms of participant demographics, the majority, 79.1% (72/91, 95% CI 69.0–86.7%) of subjects were under 6 years old, of whom 63.9% were infected (46/72, 95% CI 51.7–74.6%). Genders were almost evenly distributed, 52.7% (48/91, 95% CI 42.0–63.2%) of subjects were male and 47.3% (43/91, 95% CI 36.8–57.9%) female.

**Table 1:** Summary of included participant demographics

Demographic	Clinical (n = 16)	Community (n = 75)
Gender		
Female	6	37
Male	10	38
Age		
0–1	0	6
2–3	8	29
4–5	4	25
6–7	1	6
8–9	3	7
10–11	0	2
12–13	0	0
Education (current)		
Nursery	9	14
Primary	7	11
Secondary	0	1
Other	0	49

**Fig. 1:**
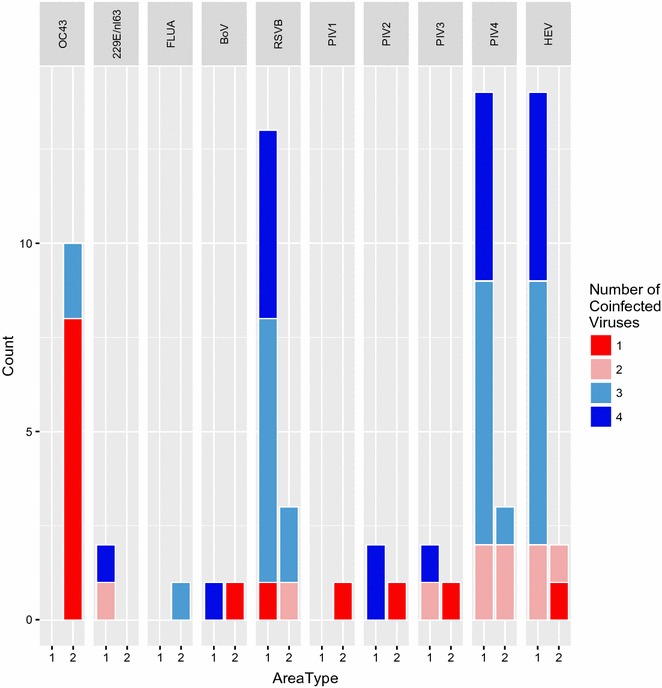
Distribution of virus types [coronavirus (OC 229 E/NL63, OC43), influenza A and B (FLUA, FLUB), bocavirus (BoV), respiratory syncytial virus (RSV) A and B, rhinovirus type A-C, and parainfluenza virus 1, 2, 3 and 4 (PIV1–4), enterovirus (HEV), adenovirus (ADV), metapneumovirus (hMPV)] and number of coinfections across all clinical (Area 1) and community (Area 2) samples

**Fig. 2:**
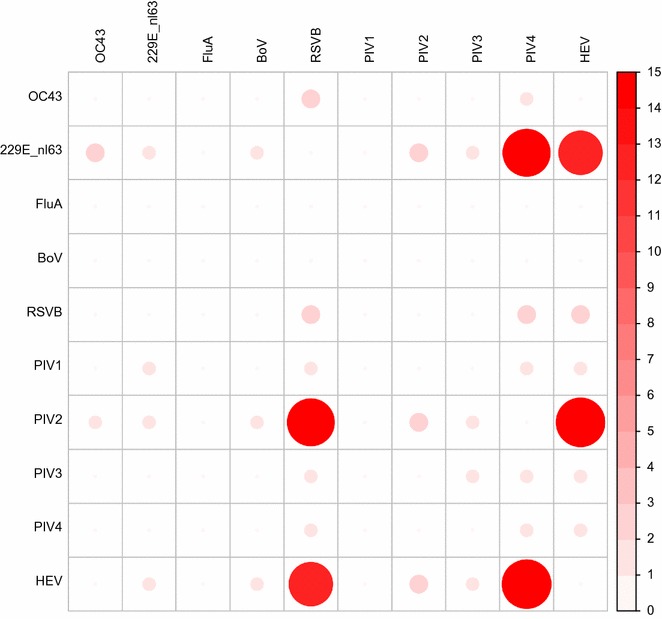
Coinfection distribution of viruses across all samples [coronavirus (OC 229 E/NL63, OC43), influenza A and B (FLUA, FLUB), bocavirus (BoV), respiratory syncytial virus (RSV) A and B, rhinovirus type A-C, and parainfluenza virus 1, 2, 3 and 4 (PIV1–4), enterovirus (HEV), adenovirus (ADV), metapneumovirus (hMPV)]. Shade and size of circle correspond to the number of persons who had both the virus corresponding to the row and column of the cell

PCR results showed that ten different viruses (influenza A, coronavirus OC 229 E/NL63, RSVA, RSV B, parainfluenza 1–4) were detected. Figure 1 shows how these infections were distributed across virus types as well as in the community versus clinic samples. In sum, a total of 33 of the 91 subjects surveyed had one or more respiratory tract virus (36.3%, 95% CI 26.6–47.0%, Fig. 1). Furthermore, 10 of those cases were triple infections and 5 were quadruple infections (illustrated by color of bars in Fig. 1). Figure 2 indicates how frequently each pair of viruses were found in the same participant; co-infections were most common among enterovirus and parainfluenza virus 4 (Fig. 2).

We also compared and contrasted the clinical and community results. Parainfluenza virus 4, respiratory syncytial virus B and enterovirus were the most common viruses found in the clinical sample. These three infections resulted in 41 viruses detected in 15 subjects clinically, and eight infections detected in five people in the community. Together they infected 94% (15/16, 95% CI 67.7–99.7%) of clinical subjects, and 7% (5/75, 95% CI 2.5–15.5%) in the community (significant difference, p < 0.001). The most common virus detected in community samples was Coronavirus OC43; this virus was detected in 13.3% (95% CI 6.9–23.6%) people in the community and not in any of the clinical samples. However a different strain, coronavirus OC 229 E/NL63 was detected in 12.5% of the clinical subjects (2/16, 95% CI 2.2–39.6%) and not detected in the community. Double, triple and quadruple infections were another common feature of note.

### Discussion

We identified ten different respiratory tract viruses among the subjects as shown in Fig. 1. Samples collected from the Children’s specialist hospital showed 100% prevalence rate of infection with one or more viruses. This might not be surprising, as the basic difference between the community and clinic samples was an increased severity of illness in the clinical sample. This may also explain the high level of co-infection found among the clinical subjects. The most prevalent virus in the clinical sample (coronavirus OC43) was not detected in the community sample. Further, there was a significant difference between prevalence of the most common viruses in the clinical sample (parainfluenza virus 4, respiratory syncytial virus B and enterovirus) and their prevalence in the community. Finally, some of the viruses detected in this study have not been detected and implicated with ARIs in Nigeria. There is no report, to the best of our knowledge, implicating coronavirus in ARIs in Nigeria, and it was detected in 12 subjects in this study. Although cases of double and triple infections were observed in a study in Nigeria in 2011 [28], as far as we are aware, reports of quadruple infections are rare and have not been reported in Nigeria previously.

Due to the unique nature of the data generated in this study and novelty of work in the setting, it is not possible to exactly compare results to other studies. For example, though we found a similar study regarding ARIs in clinical subjects in Burkina Faso [27], due to the small sample size from this study it would not be feasible to infer or compare prevalence rates. Studies of ARI etiology have mostly been generally focused in areas of the world that are more developed [29], and it is important to note that the availability of molecular diagnostic methods as employed in this study substantially improve the ability to detect viruses which hitherto have not been detected in Nigeria. Further, findings from this work also add to the growing body of research that shows value of community-data in infectious disease surveillance [8]. As most of the work to-date has been in higher resource areas of the world this study adds perspective from an area where healthcare resources are lower.

In conclusion, results of this study provide evidence for active community surveillance to enhance public health surveillance and scientific understanding of ARIs. This is not only because a minority of children with severe infection are admitted to the hospital in areas such this in Nigeria, but also findings from this pilot study which indicate that viral circulation in the community may not get detected clinically [29]. This pilot study indicates that in areas of Nigeria, etiology of ARIs ascertained from clinical samples may not represent all of the ARIs circulating in the community.

## Limitations

The main limitation of the study is the sample size. In particular, the sample is not equally representative across all ages. However, the sample size was big enough to ascertain significant differences in community and clinic sourced viruses, and provides a qualitative understanding of viral etiology in samples from the community and clinic. Moreover, the sample was largely concentrated on subjects under 6 years, who are amongst the groups at highest risk of ARIs. Despite the small sample size, samples here indicate that circulation patterns in the community may differ from those in the clinic. In addition, this study resulted in unique findings including detection of the first quadruple infection in Nigeria.

Given that resources are limited for research and practice, we hope these pilot results may motivate further systematic investigations into how community-generated data can best be used in ARI surveillance. Results of this study can inform a larger study, representative across demographic and locations to systematically assess the etiology of infection and differences in clinical and community cohorts. A larger study will also enable accounting for potential confounders such as environmental risk factors. Finally, while it may be intuitive, findings from this pilot study shed light on the scope of differences in ARI patterns including different types and strains of circulating viruses. Also, because PCR was used for viral detection, the study was limited to detection of viruses in the primer sets. Given that these are the most up-to-date and common viruses, this approach was deemed sufficient for this initial investigation.

## Abbreviations

ARI: acute respiratory infection
URI: upper respiratory tract infection
LRI: lower respiratory tract infection
HCoV-EMC: human coronavirus-Erasmus Medical Center
DNA: deoxyribonucleic acid
RNA: ribonucleic acid
PCR: polymerase chain reaction
PIV1–4: parainfluenza virus 1, 2, 3 and 4
RSV: respiratory syncytial virus
FLUA, FLUB: influenza A and B
ADV: adenovirus
HEV: enterovirus
hMPV: metapneumovirus
BoV: bocavirus
